# A comparative review of economic evaluations for immune checkpoint inhibitors in early stage and advanced stage cancer: focusing on pembrolizumab

**DOI:** 10.3389/fphar.2026.1717776

**Published:** 2026-03-23

**Authors:** Hyo-Jin Kim, Ae-Ryeo Cho, Joo-Young Byun, Eui-Kyung Lee

**Affiliations:** 1 School of Pharmacy, Sungkyunkwan University, Suwon, Republic of Korea; 2 VIAplus, Suwon, Republic of Korea; 3 Department of Surgery, Penn State College of Medicine, Hershey, PA, United States

**Keywords:** advanced stage cancer, early stage cancer, economic evaluation, health technology assessment, immune checkpoint inhibitors, pembrolizumab

## Abstract

**Introduction:**

While immune checkpoint inhibitors (ICIs) are increasingly used in early stage cancers, how economic evaluation methods differ between early and advanced stages remains underexplored. This study aimed to compare methodological differences in economic evaluations between cancer stages and examine consistency between technology appraisals (TAs) and published articles, using pembrolizumab as an example.

**Methods:**

A systematic literature review identified economic evaluations of melanoma, triple-negative breast cancer (TNBC), and renal cell carcinoma (RCC) from PubMed, Embase, and the Cochrane Library (protocol registered in PROSPERO [CRD42025646192]). Methodological characteristics and cost-effectiveness conclusions for pembrolizumab were compared between early and advanced stage articles. TAs from the National Institute for Health and Care Excellence (NICE), Canada’s Drug Agency (CDA-AMC), and Pharmaceutical Benefits Advisory Committee (PBAC) were also reviewed.

**Results:**

Forty-one articles and 25 TAs were included. Economic evaluations in early stage cancers more frequently used Markov models, longer time horizons, distinct health states for recurrence and metastasis, cure assumptions, and more often concluded pembrolizumab to be cost-effective (p < 0.05). Differences between TAs and published articles were identified in time horizon and assumptions regarding treatment effect waning. Among the published articles, methodological choices related to time horizon, utility approach, and relative dose intensity (RDI) were associated with cost-effectiveness conclusions across cancer stages.

**Conclusion:**

Methodological variations in economic evaluations of pembrolizumab were observed between early stage and advanced stage cancers in published articles, as well as between TAs and published articles across both stages. This study highlights the need for stage-specific modeling and clearer methodological guidance to support robust economic evaluations of ICIs in both early and advanced cancer settings.

## 1 Introduction

Immune checkpoint inhibitors (ICIs) block immune checkpoint proteins expressed on immune cells (e.g., T cells) and cancer cells (Alturki, 2023). Several ICIs have been approved by the U.S. Food and Drug Administration (FDA), including programmed cell death protein 1 (PD-1) inhibitors, programmed cell death ligand 1 (PD-L1) inhibitors, and cytotoxic T-lymphocyte-associated antigen 4 (CTLA-4) inhibitors (Liebl and Hofmann, 2019; Shiravand et al., 2022; Paul et al., 2024). ICIs have primarily been used in advanced or metastatic settings where curative treatment options such as surgery are not feasible. Recently, they have been increasingly adopted as adjuvant therapies in patients with early stage cancers eligible for curative treatments (Vansteenkiste et al., 2019; Ma et al., 2023; Cui et al., 2024).

Early and advanced stage cancers differ markedly with respect to disease progression, treatment approaches, and prognosis. Early stage cancer is usually managed with curative surgery and perioperative therapies, generally with a favorable prognosis, but there remains a risk of local recurrence or distant metastasis. In contrast, advanced stage cancer is generally inoperable and managed with systemic therapies alone, often resulting in poorer survival outcomes (Faubert et al., 2020; He et al., 2022; Ellison and Saint-Jacques, 2023; Alinia et al., 2024; Cuppens et al., 2024). These clinical distinctions by cancer stage suggest that different approaches may be needed for early and advanced stage cancer in health economic evaluations, in terms of model structures, time horizon, and key model assumptions.

Since the approval and reimbursement assessment of ICIs for early stage cancer in 2018–2019 (Donia et al., 2025), economic evaluations of ICIs as adjuvant therapies in early stage cancers have steadily emerged (Bensimon et al., 2019; Chen et al., 2022; Lai et al., 2023; Zhang et al., 2023; Casabianca et al., 2024). However, previous systematic reviews on the economic evaluations of ICIs have mostly focused on advanced stage cancers (Ding et al., 2020; Li et al., 2020; Zhang et al., 2022; Dawood et al., 2023; Gong et al., 2024). Accordingly, there has been a lack of reviews or methodological discussions specifically addressing economic evaluations for early stage cancers, making it difficult to distinguish stage-specific considerations.

Furthermore, there has been scarce evidence on how economic evaluations on ICIs submitted to health technology assessment (HTA) agencies (e.g., technology appraisals [TAs] in National Institute for Health and Care Excellence [NICE]) and those reported in published academic articles differ. While TAs are directly tied to reimbursement decision-making and reflect institutional priorities and evidentiary standards, academic articles may differ in their methodological choices, perspectives, or assumptions. By examining the degree of alignment between academic research and TAs, this study facilitates a better understanding of how closely academic evaluations reflect real-world policy considerations. Such comparisons may help identify opportunities to improve methodological transparency, facilitate mutual understanding between academic modeling approaches and HTA decision-making, and ultimately promote consistent approaches to economic evaluation that are tailored to disease stage and justified by clinical evidence.

Given that pembrolizumab has received regulatory approval for a wide range of indications across multiple cancer types and stages, it well suits for examining how economic evaluations vary by disease stage. Therefore, we aim to review the economic evaluations of ICIs using pembrolizumab as an example, comparing evaluations between early and advanced stages, and examining recommendations from TAs alongside the methodologies used in academic evaluations at each stage.

## 2 Methods

### 2.1 Cancer types

In this study, we selected cancer types that met both of the following criteria: (1) cancers for which pembrolizumab had received U.S. FDA approval for early stage adjuvant therapy (pre- or post-surgical) and for first or subsequent line treatment in advanced stage by July 2024; and (2) cancers that had been evaluated for both early and advanced stage treatment by at least one of the following agencies: NICE, Canada’s Drug Agency (CDA-AMC), or the Pharmaceutical Benefits Advisory Committee (PBAC).

Melanoma, non-small cell lung cancer (NSCLC), triple-negative breast cancer (TNBC), and renal cell carcinoma (RCC) had all received U.S. FDA approval for both early and advanced stage indications by July 2024. However, no TAs for early stage NSCLC had been published by NICE, CDA-AMC, or PBAC by that time, with only advanced stage data available. Therefore, melanoma, TNBC, and RCC were selected as the target cancer types for this study.

### 2.2 Systematic literature review for published economic evaluation articles

A literature search was conducted using the PubMed, Embase, and Cochrane Library databases for articles published through 3 February 2025. The search strategy was developed using keywords covering cancer types, pembrolizumab, and economic evaluations (Supplementary Tables 1-3). After removing duplicates, the remaining records were screened in two stages based on predefined inclusion and exclusion criteria. In the first screening, titles and abstracts were reviewed to exclude articles irrelevant to the study topic. In the second screening, full texts of the remaining articles were assessed, and those meeting the inclusion criteria were selected. The inclusion criteria were as follows: (1) economic evaluations conducted on the selected cancer types; (2) articles performing cost-utility analysis and reporting incremental cost-effectiveness ratios (ICERs), expressed as cost per quality-adjusted life year (QALY) gained; and (3) articles published in English. Articles were excluded if they did not report detailed methods or results, evaluated cost-effectiveness across multiple treatment sequences, or were abstracts or poster presentations. Two reviewers independently conducted the literature search and selection. Discrepancies were resolved through discussion, and if consensus was not reached, a third reviewer was consulted. This systematic literature review was conducted in accordance with the Cochrane Collaboration and Preferred Reporting Items for Systematic Reviews and Meta-Analyses (PRISMA) guidelines. The protocol was registered in PROSPERO (registration number: CRD42025646192).

To assess the methodological quality and reporting transparency of the included economic evaluation articles, we evaluated each article using the Consolidated Health Economic Evaluation Standards (CHEERS) checklist (Husereau et al., 2013; Husereau et al., 2022). Each item was independently reviewed. Articles were assessed across key domains, including study perspective, time horizon, discounting, measurement and valuation of health outcomes, handling of uncertainty, and transparency of model assumptions. The findings of this quality assessment were used to support the interpretation of the results rather than to exclude articles from the analysis.

### 2.3 Selection of technology appraisals

Details of economic evaluations reported in TAs published by the NICE, CDA-AMC, and PBAC up to July 2024 were reviewed. NICE publishes final guidance, draft guidance and committee papers, which include data submitted by manufacturers and assessments conducted by the Evidence Review Group (ERG). In this study, the committee papers were reviewed. Since 2021, CDA-AMC has revised the format of economic evaluation reports. We reviewed economic guidance reports for evaluations conducted before 2021 and integrated reports comprising clinical, pharmacoeconomic, and stakeholder inputs for those conducted thereafter. In Australia, PBAC appraisals were retrieved from public summary documents, organized by product and PBAC meeting date. Only appraisals involving the selected cancer types and using cost-utility analysis (as opposed to cost-minimization or price comparison) were included.

### 2.4 Data extraction

This study extracted only data used in the base-case analyses from both published articles and TAs, excluding data presented solely in scenario analyses. From the included published articles, the following information was extracted: cancer stage (early or advanced), indication, model type (Markov model or partitioned survival model [PSM]), health states included in the model, time horizon and perspective. Additional extracted details included treatment effect waning, cure assumption, utility approach (progression-based or time-to-death [TTD]-based), inclusion of subsequent therapy in the model, treatment duration of pembrolizumab, relative dose intensity (RDI) considered for treatment costs, and conclusions regarding the cost-effectiveness of pembrolizumab. From the included TAs, the following data were extracted: cancer stage, indication, intervention and comparators, model type, health states included in the model, time horizon, cure assumption, treatment effect waning, utility approach, RDI considered for treatment costs, whether a recommendation was made, and the timing of the recommendation. Methodological details used in the TAs were primarily identified based on the ERG’s recommendations. If the ERG did not comment on a particular methodological component, the methods described in the manufacturer’s submission were considered valid and included in the review. In both TAs and published articles, when no information was provided regarding the application of cure assumptions, treatment effect waning, or RDI, the item was classified as not applied.

### 2.5 Comparison between early stage and advanced stage cancer in published articles

Among the included published articles, we compared methodological aspects between early stage and advanced stage cancers. These aspects included model type, health states included in the model, time horizon, perspective, treatment effect waning, cure assumption, utility approach, inclusion of subsequent therapy, treatment duration of pembrolizumab, and RDI considered for treatment costs. Conclusions regarding the cost-effectiveness of pembrolizumab were also compared by stage. Statistical comparisons were conducted using the Chi-square test with a significance level of 5%. If more than 20% of the expected cell counts were below 5, Fisher’s exact test was used instead. All statistical analyses were performed using IBM SPSS statistics.

### 2.6 Comparison between published articles and TAs

Between the included published articles and TAs, we compared economic evaluation model type, health states included in the model, time horizon, and key methodological aspects frequently discussed across multiple TAs such as cure assumptions, treatment effect waning, utility approach, and RDI separately for early stage and advanced stage cancers (CDA-AMC, 2020; NICE, 2021; Hancock et al., 2022; CDA-AMC, 2023b; Trigg et al., 2024). In addition, we examined how the application of these methodological elements influenced the conclusions of pembrolizumab’s cost-effectiveness in published articles.

## 3 Results

### 3.1 Search results for published articles and TAs

**FIGURE 1:**
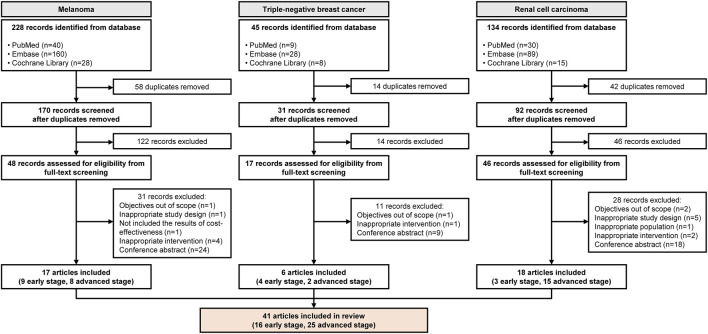
Flow chart of selecting eligible published economic evaluation articles.

A total of 228 articles on melanoma, 45 on TNBC, and 134 on RCC were identified through database searches. After applying predefined inclusion and exclusion criteria through a two-step selection process, seventeen articles for melanoma (9 early stage, 8 advanced stage), six for TNBC (4 early stage, 2 advanced stage), and eighteen for RCC (3 early stage, 15 advanced stage) were included (Figure 1). Twelve TAs for melanoma (4 from NICE, 3 from CDA-AMC, five from PBAC), seven for TNBC (2 from NICE, 2 from CDA-AMC, 3 from PBAC), and six for RCC (3 from NICE, 3 from CDA-AMC) were identified and reviewed. The characteristics of the published articles and TAs included in this study are provided in Supplementary Tables 4, 5. In addition, the included articles demonstrated moderate to high adherence to the CHEERS checklist. Detailed results of the quality assessment are presented in Supplementary Table 6.

### 3.2 Comparison between early stage and advanced stage cancer in published articles

The extracted information for the selected published articles on melanoma, TNBC, and RCC are summarized in Table 1. Regarding model types, the proportion of articles using a Markov model was higher in early stage compared with advanced stage, whereas most articles for advanced stage used a PSM (Markov model: 93.8% vs 40.0%; PSM: 0% vs 56.0%, p < 0.001). In the early stage articles, it was more common to include four or more health states, subdividing disease progression into local recurrence (LR) and distant metastasis (DM). By contrast, advanced stage articles typically used three health states, including progression-free (PF), progressed disease (PD), and death, with 100% of advanced stage articles using these states compared to 6.3% of early stage articles; 93.8% of early stage articles used four or more states (p < 0.001). Longer time horizons were generally applied in early stage articles. While articles with a time horizon of less than 20 years were more common in advanced stage compared with early stage, this difference was not statistically significant (18.8% vs 56.0%, p = 0.056). Treatment effect waning was not applied in any articles of either stage. No advanced stage articles adopted a cure assumption, whereas approximately 38% of early stage articles did, representing a statistically significant difference (p = 0.002). In the case of utility approach, TTD-based utility was used in about one-quarter of advanced stage articles, whereas no early stage articles applied this approach (p = 0.021).

**TABLE 1:** Characteristics on published articles: early stage vs advanced stage.

​	Total (melanoma, TNBC, RCC)		
	Early stage (N = 16)	Advanced stage (N = 25)	p-value
Model type	n (%)	n (%)	
Markov model	15 (93.8%)	10 (40.0%)	<0.001
PSM	0 (0%)	14 (56.0%)	​
Other^a^	1 (6.3%)	1 (4.0%)	​
No. Health state			
3 states	1 (6.3%)	25 (100%)	<0.001
≥4 states	15 (93.8%)	0 (0%)	​
Type of health state			
PF/PD/Death	1 (6.3%)	25 (100%)	<0.001
RF/LR/DM/Death	14 (87.5%)	0 (0%)	​
Other^b^	1 (6.3%)	0 (0%)	​
Time horizon			
≤20 years	3 (18.8%)	14 (56.0%)	0.056
21+ years	8 (50.0%)	6 (24.0%)	​
Lifetime (w/o period)	5 (31.3%)	4 (16.0%)	​
NA	0 (0.0%)	1 (4.0%)	​
Perspective			
Payer or Health system	13 (81.3%)	23 (92.0%)	0.362
Societal	3 (18.8%)	2 (8.0%)	​
Treatment effect waning			
Yes: at 0–5 years	0 (0%)	0 (0%)	NC
Yes: at 5+ years	0 (0%)	0 (0%)	​
No	16 (100%)	25 (100%)	​
Cure assumption			
Yes: at 0–5 years	1 (6.3%)	0 (0%)	0.002
Yes: at 5+ years	5 (31.3%)	0 (0%)	​
No	10 (62.5%)	25 (100%)	​
Utility approach			
Progression-based	16 (100%)	16 (64.0%)	0.021
TTD-based	0 (0%)	6 (24.0%)	​
Other^c^	0 (0%)	3 (12.0%)	​
Subsequent therapy			
Systemic therapy	16 (100%)	15 (60.0%)	0.003
Surgery	6 (37.5%)	0 (0%)	0.002
Radiotherapy	3 (18.8%)	0 (0%)	0.053
Best supportive care	2 (12.5%)	8 (32.0%)	0.265
Other	0 (0%)	8 (32.0%)	0.014
Treatment duration of pembrolizumab			
Until progression or up to 2 years	0 (0%)	19 (76.0%)	<0.001
1 year	9 (56.3%)	0 (0%)	​
Other	4 (25.0%)	0 (0%)	​
NA	3 (18.8%)	6 (24.0%)	​
RDI			
Yes	11 (68.8%)	4 (16.0%)	<0.001
No	5 (31.3%)	21 (84.0%)	​
Cost-effectiveness			
Cost-effective	14 (87.5%)	9 (36.0%)	0.003
Not cost-effective	2 (12.5%)	14 (56.0%)	​
Other^d^	0 (0%)	2 (8.0%)	​

DM, distant metastasis; LR, local recurrence; NA, not available; PD, progressed disease; PF, progression-free; PSM, partitioned survival model; RCC, renal cell carcinoma; RDI, relative dose intensity; RF, recurrence-free; TNBC, triple-negative breast cancer; TTD, time-to-death.

^a^ Other includes a microsimulation model.

^b^ Other refers to health states not captured under the PF/PD/Death or RF/LR/DM/Death categories. Health states labeled as event-free (EF) or disease-free (DF) were interpreted as corresponding to RF.

^c^ Other includes approaches that consider both progression-based and TTD-based utilities, as well as those applying average utility values across all health states.

^d^ Other refers to articles in which both cost-effective and not cost-effective outcomes were reported, either due to the presence of multiple comparators or because different scenarios or conditions were analyzed for a single comparator.

Surgery and radiotherapy were included as subsequent therapies only in early stage articles. Several early stage articles also reported a shorter duration of pembrolizumab treatment compared to advanced stage articles (1 year: 56.3% vs 0%; until disease progression or up to 2 years: 0% vs 76.0%, p < 0.001). In addition, a higher proportion of early stage articles applied RDI in treatment cost calculations compared to advanced stage articles (68.8% vs 16.0%, p < 0.001). Regarding cost-effectiveness conclusions, pembrolizumab was more frequently found to be cost-effective in early stage than in advanced stage (87.5% vs 36.0%, p = 0.003).

### 3.3 Comparison between published articles and TAs

#### 3.3.1 Melanoma

In early stage melanoma, all TAs and 8 of 9 published articles used a Markov model with recurrence-free (RF), LR, DM, and death as health states. Regarding the time horizon, NICE accepted 46 years and 40.7 years, while CDA-AMC and PBAC considered 5–25 years or 10 years, respectively. Most published articles, unlike CDA-AMC and PBAC recommendations, applied 46 years, 40.7 years or lifetime without specifying the duration (hereafter referred to as “lifetime”). One article, however, used a shorter 5 years-time horizon, which resulted in a non-cost-effective conclusion, in contrast to other articles using longer time horizons that reported pembrolizumab as cost-effective. NICE and CDA-AMC recommended applying a cure assumption at the 10-year time point, and most published articles also applied the cure assumption at or beyond 10 years, showing a similar pattern. Treatment effect waning was recommended by CDA-AMC and PBAC but was not used in any published articles. All TAs and published articles applied a progression-based utility approach. In terms of RDI, only NICE accepted its use in TAs, while CDA-AMC and PBAC did not recommend it. Among the published articles, five of 9 applied RDI while the remaining four did not.

For advanced stage melanoma, PSM with health states of PF, PD, and death were accepted by both NICE and PBAC, and were used in all published articles. In the context of time horizon, NICE accepted 30 years, while CDA-AMC and PBAC recommended 5–10 years. Contrary to CDA-AMC and PBAC recommendations, all published articles applied a time horizon of 20–40 years or lifetime. Cure assumptions and treatment effect waning were neither addressed nor applied in any TAs or published articles. NICE accepted a TTD-based utility approach, whereas CDA-AMC and PBAC recommended a progression-based utility approach. Among the eight published articles, four used a TTD-based utility approach, all of which concluded that pembrolizumab was cost-effective. In terms of RDI, only NICE accepted its use in TAs, while CDA-AMC and PBAC did not. Only two published articles that applied RDI demonstrated cost-effectiveness of pembrolizumab, whereas articles without RDI application more often concluded that pembrolizumab was not cost-effective (Table 2).

**TABLE 2:** Comparison of attributes reported by TAs versus published articles: Melanoma.

​	Technology appraisals (TAs)			Published articles
	NICE	CDA-AMC	PBAC	
Early stage (published articles: n = 9)				
Model type	• TA766: Markov model • TA837: Markov model	• PC0168: Markov model • PC0286: Markov model	• Markov model	• **Markov model:** (Bensimon et al., 2019)^a^, (Bensimon et al., 2020b)^a^, (Favre-Bulle et al., 2023a)^a^, (Lopez-Vinueza et al., 2023)^a^, (Mulder et al., 2021)^a^, (Standage et al., 2021; Wurcel et al., 2021)^a^, (Zhang et al., 2023)^a^ • **Microsimulation model:** (Mojtahed et al., 2021)^a^
Type of health state	• TA766: RF, LR, DM, Death • TA837: RF, LR, DM, Death	• PC0168: RF, LR, DM, Death • PC0286: RF, LR, DM, Death	• RF, LR, DM, Death	• **RF, LR, DM, Death:** (Bensimon et al., 2019)^a^, (Bensimon et al., 2020b)^a^, (Favre-Bulle et al., 2023a)^a^, (Lopez-Vinueza et al., 2023)^a^, (Mojtahed et al., 2021)^a^, (Standage et al., 2021; Wurcel et al., 2021)^a^, (Zhang et al., 2023)^a^ • **NED, RPD, Death:** (Mulder et al., 2021)^a^
Time horizon	• TA766: 46years • TA837: 40.7years	• PC0168: 5–25years • PC0286: 10years	• 10years	• **46years:** (Bensimon et al., 2019)^a^, (Lopez-Vinueza et al., 2023)^a^, (Wurcel et al., 2021)^a^ • **40.7years:** (Favre-Bulle et al., 2023a)^a^ • **19.4years:** (Mojtahed et al., 2021)^a^ • **5years:** (Standage et al., 2021) • **Lifetime (w/o period):** (Bensimon et al., 2020b)^a^, (Mulder et al., 2021)^a^, (Zhang et al., 2023)^a^
Cure assumption	• TA766: No • TA837: Yes (at 10years)	• PC0168: No • PC0286: Yes (at 10years)	• No	• **Yes (at 10+yrs):** (Bensimon et al., 2019)^a^, (Bensimon et al., 2020b)^a^, (Favre-Bulle et al., 2023a)^a^, (Lopez-Vinueza et al., 2023)^a^, (Zhang et al., 2023)^a^ • **Yes (at 2+yrs):** (Mojtahed et al., 2021)^a^ • **No:** (Mulder et al., 2021)^a^, (Standage et al., 2021; Wurcel et al., 2021)^a^
Treatment effect waning	• TA766: No • TA837: No	• PC0168: No • PC0286: Yes (at 2years)	• Yes	• **No:** (Bensimon et al., 2019)^a^, (Bensimon et al., 2020b)^a^, (Favre-Bulle et al., 2023a)^a^, (Lopez-Vinueza et al., 2023)^a^, (Mojtahed et al., 2021)^a^, (Mulder et al., 2021)^a^, (Standage et al., 2021; Wurcel et al., 2021)^a^, (Zhang et al., 2023)^a^
Utility approach	• TA766: Progression-based • TA837: Progression-based	• PC0168: Progression-based • PC0286: Progression-based	• Progression-based	• **Progression-based:** (Bensimon et al., 2019)^a^, (Bensimon et al., 2020b)^a^,(Favre-Bulle et al., 2023a)^a^, (Lopez-Vinueza et al., 2023)^a^, (Mojtahed et al., 2021)^a^, (Mulder et al., 2021)^a^, (Standage et al., 2021; Wurcel et al., 2021)^a^, (Zhang et al., 2023)^a^
RDI	• TA766: Yes • TA837: Yes	• PC0168: No • PC0286: No	• No	• **Yes:** (Bensimon et al., 2019)^a^, (Bensimon et al., 2020b)^a^, (Favre-Bulle et al., 2023a)^a^, (Lopez-Vinueza et al., 2023)^a^, (Zhang et al., 2023)^a^ • **No:** (Mojtahed et al., 2021)^a^, (Mulder et al., 2021)^a^, (Wurcel et al., 2021)^a^, (Standage et al., 2021)
Recommendation	• TA766: Recommended • TA837: Recommended	• PC0168: Recommended • PC0286: Recommended	• Recommended	-
Advanced stage (published articles: n = 8)				
Model type	• TA357: PSM • TA366: PSM	• PC0058: NA	• PSM	• **PSM:** (Ball et al., 2023; Bashari et al., 2024; Heine et al., 2024; Loong et al., 2020)^a^, (Miguel et al., 2017)^a^, (Tang et al., 2022)^a^, (Wang et al., 2017)^a^, (Wu and Shi, 2020)^a^
Type of health state	• TA357: PF, PD, Death • TA366: PF, PD, Death	• PC0058: NA	• PF, PD, Death	• **PF, PD, Death:** (Ball et al., 2023; Bashari et al., 2024; Heine et al., 2024; Loong et al., 2020)^a^, (Miguel et al., 2017)^a^, (Tang et al., 2022)^a^, (Wang et al., 2017)^a^, (Wu and Shi, 2020)^a^
Time horizon	• TA357: 30years • TA366: 30years	• PC0058: Ipi-naive 10years, Ipi-refractory 5years	• 5–10years	• **40years:** (Miguel et al., 2017)^a^ • **30years:** (Bashari et al., 2024; Heine et al., 2024; Loong et al., 2020)^a^ • **20years:** (Ball et al., 2023; Tang et al., 2022)^a^, (Wang et al., 2017)^a^ • **Lifetime (w/o period):** (Wu and Shi, 2020)^a^
Cure assumption	• TA357: No • TA366: No	• PC0058: No	• No	• **No:** (Ball et al., 2023; Bashari et al., 2024; Heine et al., 2024; Loong et al., 2020)^a^, (Miguel et al., 2017)^a^, (Tang et al., 2022)^a^, (Wang et al., 2017)^a^, (Wu and Shi, 2020)^a^
Treatment effect waning	• TA357: No • TA366: No	• PC0058: No	• No	• **No:** (Ball et al., 2023; Bashari et al., 2024; Heine et al., 2024; Loong et al., 2020)^a^, (Miguel et al., 2017)^a^, (Tang et al., 2022)^a^, (Wang et al., 2017)^a^, (Wu and Shi, 2020)^a^
Utility approach	• TA357: TTD-based • TA366: TTD-based	• PC0058: Progression-based	• Progression-based	• **Progression-based:** (Ball et al., 2023; Bashari et al., 2024; Wu and Shi, 2020)^a^ • **TTD-based:** (Loong et al., 2020)^a^, (Miguel et al., 2017)^a^, (Tang et al., 2022)^a^, (Wang et al., 2017)^a^ • **Both:** (Heine et al., 2024)
RDI	• TA357: Yes • TA366: Yes	• PC0058: No	• No	• **Yes:** (Miguel et al., 2017)^a^, (Tang et al., 2022)^a^ • **No:** (Ball et al., 2023; Bashari et al., 2024; Heine et al., 2024; Loong et al., 2020)^a^, (Wang et al., 2017)^a^, (Wu and Shi, 2020)^a^
Recommendation	• TA357: Recommended • TA366: Recommended	• PC0058: Recommended	• Recommended	-

DM, distant metastasis; Ipi, Ipilimumab; LR, local recurrence; NA, not available; NED, No evidence of disease; PD, progressed disease; PF, progression-free; PSM, partitioned survival model; RDI, relative dose intensity; RPD, recurrent/progressive disease; TAs, technology appraisals; TTD, time-to-death; RF, recurrence-free.

Reference to technical appraisals: TA766 (NICE, 2018), TA837 (NICE, 2022c), TA357 (NICE, 2015a), TA366 (NICE, 2015b), PC0168 (CDA-AMC, 2019), PC0286 (CDA-AMC, 2023b), PC0058 (CDA-AMC, 2015), PBAC (PBAC, 2015; 2016a; b; 2018; 2019).

^a^ Denotes article in which at least one evaluated regimen was found to be cost-effective.

#### 3.3.2 TNBC

In early stage TNBC, all TAs and published articles used a Markov model with health states of event-free (EF), LR, DM, and death. Regarding the time horizon, NICE and CDA-AMC accepted 51 years, while PBAC accepted 30 years. Consistent with the TA recommendations, all published articles applied a time horizon of 51 years or 32 years. Neither TAs nor published articles recommended or referenced a cure assumption. Both TAs and published articles applied a progression-based utility approach. Treatment effect waning was recommended by CDA-AMC and PBAC, but none of the published articles implemented it. In terms of RDI, it was accepted by both NICE and PBAC and applied in all published articles.

For advanced stage TNBC, all TAs adopted a PSM with health states of PF, PD, and death. Most published articles also included these three health states, although one article used a Markov model. Regarding the time horizon, NICE accepted 35 years, while CDA-AMC and PBAC recommended 10–20 years. Consistent with CDA-AMC and PBAC recommendations, both published articles applied a time horizon of 10 or 20 years. Of these, the article using a 10-year time horizon did not find pembrolizumab to be cost-effective, whereas the article with a 20-year time horizon concluded that it was cost-effective. Treatment effect waning was recommended by NICE but was not applied in any published articles. NICE accepted a TTD-based utility approach, while CDA-AMC and PBAC recommended a progression-based utility approach. Among the two published articles, the one using TTD-based utility concluded that pembrolizumab was cost-effective, whereas the other using progression-based utility found it not cost-effective. In terms of RDI, NICE accepted its use, and it was applied in one of the two articles; this articles demonstrated cost-effectiveness, while the article without RDI did not (Table 3).

**TABLE 3:** Comparison of attributes reported by TAs versus published articles: triple-negative breast cancer.

​	Technology appraisals (TAs)			Published articles
	NICE	CDA-AMC	PBAC	
Early stage (published articles: n = 4)				
Model type	• TA851: Markov model	• PC0279: Markov model	• Markov model	• **Markov model:** (Favre-Bulle et al., 2023b)^a^, (Huang et al., 2023)^a^, (Kwong et al., 2024)^a^, (Pöllinger et al., 2025)^a^
Type of health state	• TA851: EF, LR, DM, Death	• PC0279: EF, LR, DM, Death	• EF, LR, DM, Death	• **EF, LR, DM, Death:** (Favre-Bulle et al., 2023b)^a^, (Huang et al., 2023)^a^, (Kwong et al., 2024)^a^, (Pöllinger et al., 2025)^a^
Time horizon	• TA851: 51years	• PC0279: 51years	• 30years	• **51years:** (Favre-Bulle et al., 2023b)^a^, (Huang et al., 2023)^a^, (Pöllinger et al., 2025)^a^ • **32years:** (Kwong et al., 2024)^a^
Cure assumption	• TA851: No	• PC0279: No	• No	• **No:** (Favre-Bulle et al., 2023b)^a^, (Huang et al., 2023)^a^, (Kwong et al., 2024)^a^, (Pöllinger et al., 2025)^a^
Treatment effect waning	• TA851: No	• PC0279: Yes (at 3years)	• Yes (at 5years)	• **No:** (Favre-Bulle et al., 2023b)^a^, (Huang et al., 2023)^a^, (Kwong et al., 2024)^a^, (Pöllinger et al., 2025)^a^
Utility approach	• TA851: Progression-based	• PC0279: Progression-based	• Progression-based	• **Progression-based:** (Favre-Bulle et al., 2023b)^a^, (Huang et al., 2023)^a^, (Kwong et al., 2024)^a^, (Pöllinger et al., 2025)^a^
RDI	• TA851: Yes	• PC0279: No	• Yes	• **Yes:** (Favre-Bulle et al., 2023b)^a^, (Huang et al., 2023)^a^, (Kwong et al., 2024)^a^, (Pöllinger et al., 2025)^a^
Recommendation	• TA851: Recommended	• PC0279: Recommended	• Recommended	-
Advanced stage (published articles: n = 2)				
Model type	• TA801: PSM	• PC0295: PSM	• PSM	• **PSM:** (Huang et al., 2022)^a^ • **Markov model:** (Zhu et al., 2023a)
Type of health state	• TA801: PF, PD, Death	• PC0295: PF, PD, Death	• PF, PD, Death	• **PF, PD, Death:** (Huang et al., 2022)^a^, (Zhu et al., 2023a)
Time horizon	• TA801: 35years	• PC0295: 20years	• 10years	• **20years:** (Huang et al., 2022)^a^ • **10years:** (Zhu et al., 2023a)
Cure assumption	• TA801: No	• PC0295: No	• No	• **No:** (Huang et al., 2022)^a^, (Zhu et al., 2023a)
Treatment effect waning	• TA801: Yes (at 5years)	• PC0295: No	• No	• **No:** (Huang et al., 2022)^a^, (Zhu et al., 2023a)
Utility approach	• TA801: TTD-based	• PC0295: Progression-based	• Progression-based	• **Progression-based:** (Zhu et al., 2023a) • **TTD-based:** (Huang et al., 2022)^a^
RDI	• TA801: Yes	• PC0295: No	• No	• **Yes:** (Huang et al., 2022)^a^ • **No:** (Zhu et al., 2023a)
Recommendation	• TA801: Recommended	• PC0295: Recommended	• Recommended	-

DM, distant metastasis; EF, event-free; LR, local recurrence; NA, not available; PD, progressed disease; PF, progression-free; PSM, partitioned survival model; RDI, relative dose intensity; TAs, technology appraisals; TTD, time-to-death.

Reference to technical appraisals: TA851 (NICE, 2022d), TA801 (NICE, 2022a), PC0279 (CDA-AMC, 2022b), PC0295 (CDA-AMC, 2023c), PBAC (PBAC, 2023a; b; c).

^a^ Denotes article in which at least one evaluated regimen was found to be cost-effective.

#### 3.3.3 RCC

In early stage RCC, all TAs and two of three published articles used a Markov model with health states of disease-free (DF), LR, DM, and death. Regarding the time horizon, NICE and CDA-AMC accepted 41.1 years and 41.6 years, respectively. Among the published articles, one applied a 5-year time horizon, while the other two applied lifetime. The article with the 5-year time horizon did not find pembrolizumab to be cost-effective, whereas the two articles using lifetime concluded that it was cost-effective. Consistent with NICE and CDA-AMC, which did not accept a cure assumption and recommended a progression-based utility approach, none of the published articles applied cure assumption, and all adopted a progression-based utility approach. Treatment effect waning was recommended by NICE and CDA-AMC but was not used in any published articles. In terms of RDI, only NICE accepted its application. Among the three published articles, two that applied RDI demonstrated cost-effectiveness of pembrolizumab, while the remaining article without RDI did not.

For advanced stage RCC, all TAs endorsed a PSM with health states of PF, PD, and death (in CDA-AMC TAs, the PF state was described as a stable/responsive disease). Although the published articles adopted the same health states, over half used a Markov model instead of a PSM, highlighting a methodological difference between TAs and published articles. Regarding the time horizon, NICE accepted 40 years, whereas CDA-AMC recommended 15 or 30 years. Published articles applied a range of time horizons from 5 years to 39 years or lifetime. The treatment effect waning was recommended by both NICE and CDA-AMC but was not implemented in any published articles. In terms of utility approach, NICE adopted a TTD-based approach, while CDA-AMC recommended a progression-based approach. Of 15 published articles, only one used a TTD-based utility approach and concluded that pembrolizumab was cost-effective. In contrast, among the remaining 13 articles using a progression-based or both approaches, the majority did not find pembrolizumab cost-effective. Regarding RDI, only NICE accepted its use, and it was applied in one published article, in which pembrolizumab was found to be cost-effective (Table 4).

**TABLE 4:** Comparison of attributes reported by TAs versus published articles: renal cell carcinoma.

​	Technologies appraisals (TAs)			Published articles
	NICE	CDA-AMC	PBAC	
Early stage (published articles: n = 3)				
Model type	• TA830: Markov model	• PC0273: Markov model	-	• **Markov model:** (Lai et al., 2023)^a^, (Schur et al., 2024)^a^, (Sharma et al., 2023)
Type of health state	• TA830: DF, LR, DM, Death	• PC0273: DF, LR, DM, Death	-	• **DF, LR, DM, Death:** (Lai et al., 2023)^a^, (Schur et al., 2024)^a^ • **No progression, Minor toxicity, Major toxicity, Pembrolizumab discontinuation, Cancer progression, Death:** (Sharma et al., 2023)
Time horizon	• TA830: 41.1yrs	• PC0273: 41.6years	-	• **5years:** (Sharma et al., 2023) • **Lifetime (w/o period):** (Lai et al., 2023)^a^, (Schur et al., 2024)^a^
Cure assumption	• TA830: No	• PC0273: No	-	• **No:** (Lai et al., 2023)^a^, (Schur et al., 2024)^a^, (Sharma et al., 2023)
Treatment effect waning	• TA830: Yes (at 4years)	• PC0273: Yes (at 4years)	-	• **No:** (Lai et al., 2023)^a^, (Schur et al., 2024)^a^, (Sharma et al., 2023)
Utility approach	• TA830: Progression-based	• PC0273: Progression-based	-	• **Progression-based:** (Lai et al., 2023)^a^, (Schur et al., 2024)^a^, (Sharma et al., 2023)
RDI	• TA830: Yes	• PC0273: No	-	• **Yes:** (Lai et al., 2023)^a^, (Schur et al., 2024)^a^ • **No:** (Sharma et al., 2023)
Recommendation	• TA830: Recommended	• PC0273: Recommended	-	-
Advanced stage (published articles: n = 15)				
Model type	• TA650: PSM • TA858: PSM	• PC0185: PSM • PC0268: PSM	-	• **PSM:** (Bensimon et al., 2020a)^a^, (Heine et al., 2024; Wang et al., 2022; Xander et al., 2023; Yoo et al., 2023) • **Markov model:** (Chan et al., 2022; Chen et al., 2019; Ding et al., 2021)^a^, (Gupta et al., 2023; Shay et al., 2021)^a^, (Su et al., 2020; Zheng et al., 2025; Zhu et al., 2020; Zhu et al., 2023b)^a^ • **Microsimulation model:** (Watson et al., 2020)
Type of health state	• TA650: PF, PD, Death • TA858: PF, PD, Death	• PC0185: Stable/Responsive disease, PD, Death • PC0268: PF, PD, Death	-	• **PF, PD, Death:** (Bensimon et al., 2020a)^a^, (Chan et al., 2022; Chen et al., 2019; Ding et al., 2021)^a^, (Gupta et al., 2023; Heine et al., 2024; Shay et al., 2021)^a^, (Su et al., 2020; Wang et al., 2022; Watson et al., 2020; Xander et al., 2023; Yoo et al., 2023; Zheng et al., 2025; Zhu et al., 2020; Zhu et al., 2023b)^a^
Time horizon	• TA650: 40years • TA858: 40years	• PC0185: 15years • PC0268: 30years	-	• **39years:** (Bensimon et al., 2020a)^a^ • **30years:** (Heine et al., 2024) • **20years:** (Chan et al., 2022; Zhu et al., 2020; Zhu et al., 2023b)^a^ • **15years:** (Xander et al., 2023) • **10years:** (Shay et al., 2021)^a^, (Su et al., 2020; Yoo et al., 2023; Zheng et al., 2025) • **5years:** (Wang et al., 2022) • **Lifetime (w/o period):** (Chen et al., 2019; Ding et al., 2021)^a^, (Gupta et al., 2023) • **NA:** (Watson et al., 2020)
Cure assumption	• TA650: No • TA858: No	• PC0185: No • PC0268: No	-	• **No:** (Bensimon et al., 2020a)^a^, (Chan et al., 2022; Chen et al., 2019; Ding et al., 2021)^a^, (Gupta et al., 2023; Heine et al., 2024; Shay et al., 2021)^a^, (Su et al., 2020; Wang et al., 2022; Watson et al., 2020; Xander et al., 2023; Yoo et al., 2023; Zheng et al., 2025; Zhu et al., 2020; Zhu et al., 2023b)^a^
Treatment effect waning	• TA650: Yes (at 5years) • TA858: No	• PC0185: Yes (at 5years) • PC0268: No	-	• **No:** (Bensimon et al., 2020a)^a^, (Chan et al., 2022; Chen et al., 2019; Ding et al., 2021)^a^, (Gupta et al., 2023; Heine et al., 2024; Shay et al., 2021)^a^, (Su et al., 2020; Wang et al., 2022; Watson et al., 2020; Xander et al., 2023; Yoo et al., 2023; Zheng et al., 2025; Zhu et al., 2020; Zhu et al., 2023b)^a^
Utility approach	• TA650: TTD-based • TA858: TTD-based	• PC0185: Progression-based • PC0268: Progression-based	-	• **Progression-based:** (Chan et al., 2022; Chen et al., 2019; Ding et al., 2021)^a^, (Gupta et al., 2023; Su et al., 2020; Wang et al., 2022; Watson et al., 2020; Xander et al., 2023; Yoo et al., 2023; Zheng et al., 2025; Zhu et al., 2020; Zhu et al., 2023b)^a^ • **TTD-based:** (Bensimon et al., 2020a)^a^ • **Both:** (Heine et al., 2024) • **Other:** (Shay et al., 2021)^a^ ^,^ ^b^
RDI	• TA650: Yes • TA858: Yes	• PC0185: No • PC0268: No	-	• **Yes:** (Bensimon et al., 2020a)^a^ • **No:** (Chan et al., 2022; Chen et al., 2019; Ding et al., 2021)^a^, (Gupta et al., 2023; Heine et al., 2024; Shay et al., 2021)^a^, (Su et al., 2020; Wang et al., 2022; Watson et al., 2020; Xander et al., 2023; Yoo et al., 2023; Zheng et al., 2025; Zhu et al., 2020; Zhu et al., 2023b)^a^
Recommendation	• TA650: Not recommended • TA858: Recommended	• PC0185: Recommended • PC0268: Recommended	-	-

DF, disease-free; DM, distant metastasis; LR, local recurrence; NA, not available; PD, progressed disease; PF, progression-free; PSM, partitioned survival model; RDI, relative dose intensity; TAs, technology appraisals; TTD, time-to-death.

Reference to technical appraisals: TA830 (NICE, 2022b), TA650 (NICE, 2020), TA858 (NICE, 2022e), PC0273 (CDA-AMC, 2023a), PC0185 (CDA-AMC, 2020), PC0268 (CDA-AMC, 2022a).

^a^ Denotes article in which at least one evaluated regimen was found to be cost-effective.

^b^ Average utilities were used at all health states.

## 4 Discussion

As the first study to compare economic evaluations of ICIs between early and advanced stage cancers, and to examine differences between TAs and published articles, our study provides a systematic comparison of modeling approaches, key assumptions, and methodological choices across cancer stages and evidence sources.

Although PSMs are commonly used in economic evaluations of treatments for advanced cancers (Woods et al., 2017), their application in early stage cancers is rare. In our review, none of the identified TAs or published articles for early stage cancers recommended or employed a PSM. Instead, most studies used Markov models, likely reflecting differences in disease characteristics and data maturity. Unlike PSMs, which derive health state occupancy directly from survival curves, Markov models simulate transitions between discrete health states based on transition probabilities. This allows for more flexible and detailed modeling of long-term disease progression (Bullement et al., 2019). In early stage cancers, where survival data are often immature and the disease course includes extended disease-free periods and the possibility of late recurrence (Faries et al., 2013; Wangchinda and Ithimakin, 2016; Batteson et al., 2018; Thomas et al., 2022), Markov models could provide a more appropriate framework than PSMs by explicitly representing distinct health states and transitions relevant to curative treatment pathways.

Time horizon in economic evaluations can substantially influence the results of analyses (Kim et al., 2017; Sharma et al., 2024). In this study, we observed that both TAs and published articles for early stage cancers generally adopted longer time horizons than those for advanced stage cancers. This difference likely reflects the substantial variation in survival outcomes by cancer stage. According to the American Cancer Society, 5-year relative survival rates for patients diagnosed with melanoma, kidney cancer, and breast cancer between 2014 and 2020 were approximately 75%–99% for localized or regional disease, compared to 18%–35% for metastatic disease (ADC, 2025a; b; c). Given these differences, longer time horizons may be necessary to adequately capture the long-term costs and benefits associated with early stage cancers. In addition to these between-stage differences, we observed a consistent pattern within each stage, where articles adopting longer time horizons tended to report more favorable cost-effectiveness outcomes. Consistent with these observations, a systematic review examining the impact of time horizon on the results of economic evaluations found that longer time horizons were generally associated with lower ICERs (Kim et al., 2017), underscoring the importance of the appropriate alignment of the time horizon with the clinical context. Separately, time horizon accepted or recommended varied across HTA agencies. Among the three HTA agencies, NICE generally accepted longer time horizons across most cancer types, whereas CDA-AMC and PBAC tended to recommend more conservative time horizons, particularly for advanced stage cancers. A previous review of oncology treatment assessments by NICE, CDA-AMC, and PBAC between 2019 and 2021 found that manufacturers’ proposed time horizons were criticized less frequently by NICE than by CDA-AMC and PBAC (Ball et al., 2022). This finding aligns with the pattern identified in our analysis, further suggesting that NICE adopts a more flexible stance regarding the choice of time horizon. Time horizons also differed by cancer type, with TNBC having the longest in early stage cancers and RCC in advanced stage cancers. These patterns likely reflect survival outcomes observed in clinical trials. Specifically, the 3-year event-free survival rate in early stage TNBC was 84.5% (Schmid et al., 2022), the highest among the three cancer types. In advanced stage RCC, overall survival was not reached within approximately 31–34 months of follow-up (Powles et al., 2020; Choueiri et al., 2023), suggesting a substantial survival benefit. Collectively, these clinical trial results may have critically influenced the choice of time horizon in economic evaluations.

Treatment effect waning is increasingly discussed in technology assessments of ICIs with stopping rules, such as pembrolizumab, as it represents a key source of uncertainty in economic evaluations and decision-making (Kamgar et al., 2022). In the present study, assumptions of treatment effect waning were more frequently recommended in TAs for early stage cancers than for advanced stage cancers. This difference may partly reflect variations in treatment duration and time horizon between the two stages. Pembrolizumab is typically administered for up to 2 years in advanced stage cancers, whereas the approved treatment duration in early stage cancers is limited to 1 year. In addition, the time horizons applied in early stage cancer were often longer than those in advanced stage, reflecting expectations for prolonged survival. Economic evaluations of pembrolizumab in early stage cancers that combine shorter treatment durations with longer time horizons may implicitly assume extended periods of benefit after treatment discontinuation. This prolonged post-treatment benefit increases uncertainty and may not be fully supported by clinical evidence. Given the limited availability of long-term survival data in many oncology indications, assumptions regarding treatment effect waning should be grounded in robust clinical evidence (Taylor et al., 2024). Furthermore, scenario analyses exploring alternative waning assumptions can help mitigate the risk of under- or overestimating treatment benefits (Latimer, 2011; Taylor et al., 2024).

None of the published articles included in this review considered the treatment effect waning assumption for either early or advanced stage cancers. A recent review of NICE assessments between 2021 and 2023 reported that incorporating treatment effect waning generally produced more conservative estimates by attenuating the clinical benefits of interventions (Trigg et al., 2024). In this context, the absence of such assumptions in published articles suggests that their findings should be interpreted with caution, particularly given the potential impact of sustained effect assumptions on cost-effectiveness outcomes. Limited data availability or uncertainty surrounding the clinical evidence may have contributed to the omission of treatment effect waning assumptions. In the absence of detailed justification in published articles, future economic evaluations of ICIs with stopping rules should employ transparent and evidence-based methods to model the duration of treatment effects.

The TTD approach, which applies utility values based on the time remaining until death (e.g., less than 30 days, 30–179 days, 180–359 days, more than 360 days), has been increasingly accepted in NICE evaluations of ICIs (Hatswell et al., 2024). While the application of TTD-based utilities requires data sources that capture utility values based on proximity to death, real-world studies providing such data remain scarce. Consequently, TTD-based utilities are often derived from patient-level data collected in clinical trials. All articles included in our review that applied TTD-based utilities were supported by pharmaceutical companies, likely facilitating access to patient-level utility data linked to time to death. In contrast, articles using progression-based utilities were generally conducted by governmental or academic institutions, or without any external sponsorship. Within early stage cancers, all published articles applied progression-based utilities, precluding the assessment of alternative utility specifications on cost-effectiveness outcomes. For advanced stage cancers, articles applying TTD-base utilities consistently reported more favorable cost-effectiveness conclusions for pembrolizumab compared with those using progression-based utilities. These observations suggest that the choice of utility approach in advanced stage cancers may have contributed to more favorable outcomes. Previous research also indicated that using TTD-based utilities can lower ICERs by 13%–30% compared with progression-based utilities in ICIs evaluations (Heine et al., 2024). This effect may be explained by the methodological characteristics of TTD-based utilities, which assign higher utility values during periods further from death, even after disease progression (Hatswell et al., 2014). Consequently, TTD-based utilities may yield greater QALY gains and lower ICERs compared to progression-based utilities under similar clinical conditions. Despite these potential benefits, HTA agencies have expressed concerns regarding TTD-based utilities, particularly their ability to capture changes in health states related to disease progression or treatment modifications, as highlighted in the CDA-AMC evaluation of pembrolizumab for advanced TNBC (CDA-AMC, 2023c). These concerns underscore the need for transparency and validation of utility modeling methods to ensure robust and credible economic evaluations in decision-making processes.

The concept of cure assumptions was only applied in articles on early stage cancers, as none of the articles in advanced stage cancers applied or discussed such assumptions. However, among the articles on early stage, we did not observe a consistent pattern in terms of whether the application of a cure assumption was associated with favorable or unfavorable cost-effectiveness conclusions. This highlights that the impact of cure assumptions appears to be influenced by interactions with other modeling choices, such as time horizon and utility approach, rather than acting as an independent determinant. By contrast, articles applying RDI consistently reported pembrolizumab as cost-effective across both early and advanced stages, whereas articles which did not apply RDI showed both cost-effective and non-cost-effective conclusions. These findings indicate that the application of RDI is associated with more favorable cost-effectiveness conclusions and may represent an important methodological consideration in economic evaluations of ICIs.

Meanwhile, the control of brain metastases represents a clinically and economically meaningful outcome that is not adequately addressed in most existing economic evaluations of ICIs (Chen et al., 2025). Brain metastases are associated not only with substantial neurological morbidity, reduced health-related quality of life, and poor prognosis, but also with a considerable economic burden driven by increased use of neuroimaging, hospitalizations, intracranial local therapies such as radiotherapy or surgery, and ongoing supportive care (Wong et al., 2008; Lv et al., 2021; Medikonda et al., 2021; Shim et al., 2022; Tawbi et al., 2022; Winther et al., 2025). These healthcare resource utilization patterns often differ substantially between patients with and without brain metastases and may meaningfully influence cost-effectiveness outcomes. Although none of the economic evaluations identified in this review explicitly modeled intracranial disease control, previous studies have suggested that failing to account for brain metastasis status may lead to an underestimation of both disease burden and treatment value (Shim et al., 2024; Chen et al., 2025). Future economic evaluations of ICIs, including PD-1 inhibitors such as pembrolizumab, may therefore benefit from more explicitly incorporating intracranial disease control into model structures or outcome assessments to better reflect clinical outcomes, quality of life, and healthcare resource use.

This study has several limitations. First, the number of published articles on early stage cancers included in our analysis was limited compared to those on advanced stage cancers, which may have prevented a sufficiently detailed comparison between the two stages. For instance, in RCC, only 3 articles addressed early stage disease compared to 15 on advanced stage disease, which may constrain the ability to draw comprehensive conclusions regarding the economic evaluation of ICIs in early stage cancers. Nevertheless, by including all available data, this study provides preliminary insights that can inform future, more detailed comparisons as further evidence emerges. Second, this review was limited by the range of cancer types included. At the time of study design, cancer indications were deliberately selected to ensure the availability of a sufficiently accumulated evidence base that allowed a structured and internally consistent comparison of economic evaluation methodologies between early and advanced stage disease, drawing on both published articles and HTA appraisals. Consequently, certain tumor types for which early stage indications or HTA evaluations were still emerging could not be included. Future studies would be able to provide a more comprehensive and generalizable understanding of the economic evaluations of ICIs by including a broader range of cancer types. Third, assumptions regarding treatment effect duration were occasionally embedded within survival models without being explicitly labeled as treatment waning. In such cases, interpretation relied on contextual cues inferred from the sources. To mitigate this, full texts and supplementary materials were thoroughly reviewed to ensure consistency and reduce the potential for misclassification. Finally, this study did not differentiate between patient subgroups within early stage or advanced stage cancers. For example, we did not distinguish between neoadjuvant or adjuvant therapy in early stage disease, nor did we consider treatment lines (first line vs subsequent line) or cancer subtypes/genetic mutations in advanced stage disease. To maximize the inclusion of all available data sources, further stratification of patient subgroups was not conducted. This limitation restricts the ability to assess potential differences in economic evaluation outcomes according to treatment settings and individual clinical profiles. Future studies with sufficient data may allow more accurate comparisons by patient subgroup.

## 5 Conclusion

This study identified methodological differences in economic evaluations of ICIs using pembrolizumab as an example between early and advanced stage cancers, particularly in model structure, time horizon, and assumptions regarding treatment effect waning. These findings highlight the importance of stage-specific modeling approaches and guidance on key assumptions in economic evaluations of ICIs. In addition, many published articles employed methodological choices that differed from the recommendations of TAs, especially regarding time horizon and treatment effect waning. These discrepancies underline the need for clearer and more consistent guidance to support robust and reliable economic evaluations for ICIs. The findings of this study are expected to serve as valuable evidence to inform decision-making in future economic evaluations and policy development for ICIs, considering differences in therapeutic positioning and evaluation context.
