# Supplementary material for: A comparative review of economic evaluations for immune checkpoint inhibitors in early stage and advanced stage cancer: focusing on pembrolizumab
Source: Front Pharmacol. 2026 Mar 23;17:1717776. doi: 10.3389/fphar.2026.1717776 (PMC13050920; doi:10.3389/fphar.2026.1717776)
Supplement: Supplementary file 1 [file Supplementaryfile1.docx]

**Supplementary Material: A Comparative Review of Economic Evaluations for Immune Checkpoint Inhibitors in Early Stage and Advanced Stage Cancer: Focusing on Pembrolizumab**

**Authors**

Hyo-Jin Kim, Ph.D.;^1,2†^ Ae-Ryeo Cho, MS;^1†^ Joo-Young Byun, Pharm.D., Ph.D.;^1,3*^ Eui-Kyung Lee, Ph.D.^1*^

^1^ School of Pharmacy, Sungkyunkwan University, Suwon, Republic of Korea

^2^ VIAplus, Suwon, Republic of Korea

^3^ Department of Surgery, Penn State College of Medicine, Hershey, Pennsylvania, USA

† Hyo-Jin Kim and Ae-Ryeo Cho contributed equally to this work as co-first authors.

* Joo-Young Byun and Eui-Kyung Lee contributed equally to this work as co-corresponding authors.

**Corresponding authors**

Eui-Kyung Lee

Address: 2066 Seoburo Jangan-gu, Suwon-si, Gyeonggi-do, Republic of Korea 16419

Email: [ekyung@skku.edu](mailto:ekyung@skku.edu)

Telephone: +82-299-4378

Joo-Young Byun

Address: 500 University Drive, Hershey, PA, USA 17033-0850

Email: jbyun@pennstatehealth.psu.edu

Telephone: +1-223-237-6995

Supplementary Table 1. Search strategies: Melanoma

| Number | Search term | Search results |
| --- | --- | --- |
| Search date: 3 February 2025 | | |
| PubMed | | |
| 1 | Melanoma[MeSH] | 114,379 |
| 2 | Melanoma[tiab] OR Melanomas[tiab] | 149,777 |
| 3 | #1 OR #2 | 167,390 |
| 4 | Pembrolizumab[Supplementary Concept] | 4,740 |
| 5 | Pembrolizumab[tiab] OR Lambrolizumab[tiab] | 10,627 |
| 6 | SCH-900475[tiab] OR SCH900475[tiab] OR “SCH 900475”[tiab] | 2 |
| 7 | MK-3475[tiab] OR MK3475[tiab] OR “MK 3475”[tiab] | 66 |
| 8 | Keytruda[tiab] | 182 |
| 9 | #4 OR #5 OR #6 OR #7 OR #8 | 11,395 |
| 10 | “Cost-Benefit Analysis”[MeSH] | 96,780 |
| 11 | Cost-Effectiveness[tiab] OR “Cost Effectiveness”[tiab] | 85,678 |
| 12 | Cost-Utility[tiab] OR “Cost Utility”[tiab] | 7,107 |
| 13 | “Economic Evaluation”[tiab] | 14,838 |
| 14 | #10 OR #11 OR #12 OR #13 | 146,520 |
| 15 | #3 AND #9 AND #14 | 40 |
| 16 | #15 Filters: English | 40 |
| Embase | | |
| 1 | ‘Melanoma’/exp | 222,725 |
| 2 | Melanoma:ab,ti OR Melanoma:ab,ti | 211,304 |
| 3 | #1 OR #2 | 268,405 |
| 4 | ‘Pembrolizumab’/exp | 47,078 |
| 5 | Pembrolizumab:ab,ti OR Lambrolizumab:ab,ti | 23,241 |
| 6 | SCH900475;ab,ti OR ‘SCH 900475’:ab,ti | 1 |
| 7 | MK3475:ab,ti OR ‘MK 3475’:ab,ti | 304 |
| 8 | Keytruda:ab,ti | 376 |
| 9 | #4 OR #5 OR #6 OR #7 OR #8 | 48,635 |
| 10 | ‘Cost Benefit Analysis’/exp | 98,636 |
| 11 | ‘Cost Effectiveness’:ab,ti | 118,166 |
| 12 | ‘Cost Utility’:ab,ti | 10,412 |
| 13 | ‘Economic Evaluation’:ab,ti | 18,034 |
| 14 | #10 OR #11 OR #12 OR #13 | 212,024 |
| 15 | #3 AND #9 AND #14 | 160 |
| Cochrane Library | | |
| 1 | MeSH descriptor: [Melanoma] explode all trees | 2,801 |
| 2 | (Melanoma OR Melanomas):ti,ab,kw | 7,077 |
| 3 | #1 OR #2 | 7,082 |
| 4 | (Pembrolizumab OR Lambrolizumab):ti,ab,kw | 3,804 |
| 5 | (SCH-900475 OR SCH900475 OR “SCH 900475”):ti,ab,kw | 12 |
| 6 | (MK-3475 OR MK3475 OR “MK 3475”):ti,ab,kw | 421 |
| 7 | (Keytruda):ti,ab,kw | 252 |
| 8 | #4 OR #5 OR #6 OR #7 | 3,830 |
| 9 | MeSH descriptor: [Cost-Benefit Analysis] explode all trees | 11,755 |
| 10 | (Cost-Effectiveness OR “Cost Effectiveness”):ti,ab,kw | 28,030 |
| 11 | (Cost-Utility OR “Cost Utility”):ti,ab,kw | 2,866 |
| 12 | (“Economic Evaluation”):ti,ab,kw | 5,453 |
| 13 | #9 OR #10 OR #11 OR #12 | 33,696 |
| 14 | #3 AND #8 AND #13 | 28 |

Supplementary Table 2. Search strategies: triple-negative breast cancer

| Number | Search term | Search results |
| --- | --- | --- |
| Search date: 3 February 2025 | | |
| PubMed | | |
| 1 | “Triple Negative Breast Neoplasms”[MeSH] | 12,634 |
| 2 | “ER-Negative PR-Negative HER2-Negative”[tiab] OR “ER Negative PR Negative HER2 Negative”[tiab] OR Triple-Negative[tiab] OR “Triple Negative”[tiab] | 27,449 |
| 3 | “Breast Neoplasm”[tiab] OR “Breast Neoplasms”[tiab] OR “Breast Cancer”[tiab] | 377,944 |
| 4 | #2 AND #3 | 26,108 |
| 5 | #1 OR #4 | 26,918 |
| 6 | Pembrolizumab[Supplementary Concept] | 4,740 |
| 7 | Pembrolizumab[tiab] OR Lambrolizumab[tiab] | 10,627 |
| 8 | SCH-900475[tiab] OR SCH900475[tiab] OR “SCH 900475”[tiab] | 2 |
| 9 | MK-3475[tiab] OR MK3475[tiab] OR “MK 3475”[tiab] | 66 |
| 10 | Keytruda[tiab] | 182 |
| 11 | #6 OR #7 OR #8 OR #9 OR #10 | 11,395 |
| 12 | “Cost-Benefit Analysis”[MeSH] | 96,780 |
| 13 | Cost-Effectiveness[tiab] OR “Cost Effectiveness”[tiab] | 85,678 |
| 14 | Cost-Utility[tiab] OR “Cost Utility”[tiab] | 7,107 |
| 15 | “Economic Evaluation”[tiab] | 14,838 |
| 16 | #12 OR #13 OR #14 OR #15 | 146,520 |
| 17 | #5 AND #11 AND #16 | 9 |
| 18 | #17 Filters: English | 9 |
| Embase | | |
| 1 | ‘Triple Negative Breast Cancer’/exp | 44,182 |
| 2 | (‘ER-Negative PR-Negative HER2-Negative’ OR ‘ER Negative PR Negative HER2 Negative’ OR ‘Triple Negative’):ab,ti | 48,572 |
| 3 | (‘Breast Neoplasm’ OR ‘Breast Neoplasms’ OR ‘Breast Cancer’):ab,ti | 533,139 |
| 4 | #2 AND #3 | 45,193 |
| 5 | #1 OR #4 | 56,002 |
| 6 | ‘Pembrolizumab’/exp | 47,078 |
| 7 | Pembrolizumab:ab,ti OR Lambrolizumab:ab,ti | 23,241 |
| 8 | SCH900475;ab,ti OR ‘SCH 900475’:ab,ti | 1 |
| 9 | MK3475:ab,ti OR ‘MK 3475’:ab,ti | 304 |
| 10 | Keytruda:ab,ti | 376 |
| 11 | #6 OR #7 OR #8 OR #9 OR #10 | 48,635 |
| 12 | ‘Cost Benefit Analysis’/exp | 98,636 |
| 13 | ‘Cost Effectiveness’:ab,ti | 118,166 |
| 14 | ‘Cost Utility’:ab,ti | 10,412 |
| 15 | ‘Economic Evaluation’:ab,ti | 18,034 |
| 16 | #12 OR #13 OR #14 OR #15 | 212,024 |
| 17 | #5 AND #11 AND #16 | 28 |
| Cochrane Library | | |
| 1 | MeSH descriptor: [Triple Negative Breast Neoplasms] explode all trees | 566 |
| 2 | (“ER-Negative PR-Negative HER2-Negative” OR “ER Negative PR Negative HER2 Negative” OR Triple-Negative OR “Triple Negative”):ti,ab,kw | 2,469 |
| 3 | (“Breast Neoplasm” OR “Breast Neoplasms” OR “Breast Cancer”):ti,ab,kw | 47,306 |
| 4 | #2 AND #3 | 2,431 |
| 5 | #1 OR #4 | 2,431 |
| 6 | (Pembrolizumab OR Lambrolizumab):ti,ab,kw | 3,804 |
| 7 | (SCH-900475 OR SCH900475 OR “SCH 900475”):ti,ab,kw | 12 |
| 8 | (MK-3475 OR MK3475 OR “MK 3475”):ti,ab,kw | 421 |
| 9 | (Keytruda):ti,ab,kw | 252 |
| 10 | #6 OR #7 OR #8 OR #9 | 3,830 |
| 11 | MeSH descriptor: [Cost-Benefit Analysis] explode all trees | 11,755 |
| 12 | (Cost-Effectiveness OR “Cost Effectiveness”):ti,ab,kw | 28,030 |
| 13 | (Cost-Utility OR “Cost Utility”):ti,ab,kw | 2,866 |
| 14 | (“Economic Evaluation”):ti,ab,kw | 5,453 |
| 15 | #11 OR #12 OR #13 OR #14 | 33,696 |
| 16 | #5 AND #10 AND #15 | 8 |

Supplementary Table 3. Search strategies: renal cell carcinoma

| Number | Search term | Search results |
| --- | --- | --- |
| Search date: 3 February 2025 | | |
| PubMed | | |
| 1 | “Carcinoma, Renal Cell’[MeSH] | 43,283 |
| 2 | Renal[tiab] OR “Renal Cell”[tiab] OR Nephroid[tiab] OR Hypernephroid[tiab] OR Kidney[tiab] | 1,051,804 |
| 3 | Carcinoma[tiab] OR Carcinomas[tiab] OR Adenocarcinoma[tiab] OR Adenocarcinomas[tiab] OR Cancer[tiab] OR Cancers[tiab] | 2,994,055 |
| 4 | #2 AND #3 | 123,892 |
| 5 | Hypernephroma[tiab] OR Hypernephromas[tiab] OR “Grawitz Tumor”[tiab] | 1,453 |
| 6 | #1 OR #4 OR #5 | 129,081 |
| 7 | Pembrolizumab[Supplementary Concept] | 4,740 |
| 8 | Pembrolizumab[tiab] OR Lambrolizumab[tiab] | 10,627 |
| 9 | SCH-900475[tiab] OR SCH900475[tiab] OR “SCH 900475”[tiab] | 2 |
| 10 | MK-3475[tiab] OR MK3475[tiab] OR “MK 3475”[tiab] | 66 |
| 11 | Keytruda[tiab] | 182 |
| 12 | #7 OR #8 OR #9 OR #10 OR #11 | 11,395 |
| 13 | “Cost-Benefit Analysis”[MeSH] | 96,780 |
| 14 | Cost-Effectiveness[tiab] OR “Cost Effectiveness”[tiab] | 85,678 |
| 15 | Cost-Utility[tiab] OR “Cost Utility”[tiab] | 7,107 |
| 16 | “Economic Evaluation”[tiab] | 14,838 |
| 17 | #13 OR #14 OR #15 OR #16 | 146,520 |
| 18 | #6 AND #12 AND #17 | 30 |
| 19 | #18 Filters: English | 30 |
| Embase | | |
| 1 | ‘Renal Cell Carcinoma’/exp | 46,406 |
| 2 | Renal:ab,ti OR ‘Renal Cell’:ab,ti OR Nephroid:ab,ti OR Hypernephroid:ab,ti OR Kidney: ab,ti | 1,511,406 |
| 3 | Carcinoma:ab,ti OR Carcinomas:ab,ti OR Adenocarcinoma:ab,ti OR Adenocarcinomas:ab,ti OR Cancer:ab,ti OR Cancers:ab,ti | 4,156,410 |
| 4 | #2 AND #3 | 190,794 |
| 5 | Hypernephroma:ab,ti OR Hypernephromas:ab,ti OR ‘Grawitz Tumor’:ab,ti | 1,612 |
| 6 | #1 OR #4 OR #5 | 206,131 |
| 7 | ‘Pembrolizumab’/exp | 47,078 |
| 8 | Pembrolizumab:ab,ti OR Lambrolizumab:ab,ti | 23,241 |
| 9 | SCH900475;ab,ti OR ‘SCH 900475’:ab,ti | 1 |
| 10 | MK3475:ab,ti OR ‘MK 3475’:ab,ti | 304 |
| 11 | Keytruda:ab,ti | 376 |
| 12 | #7 OR #8 OR #9 OR #10 OR #11 | 48,635 |
| 13 | ‘Cost Benefit Analysis’/exp | 98,636 |
| 14 | ‘Cost Effectiveness’:ab,ti | 118,166 |
| 15 | ‘Cost Utility’:ab,ti | 10,412 |
| 16 | ‘Economic Evaluation’:ab,ti | 18,034 |
| 17 | #13 OR #14 OR #15 OR #16 | 212,024 |
| 18 | #6 AND #12 AND #17 | 89 |
| Cochrane Library | | |
| 1 | MeSH descriptor: [Carcinoma, Renal Cell] explode all trees | 1,584 |
| 2 | (Renal OR “Renal Cell” OR Nephroid OR Hypernephroid OR Kidney):ti,ab,kw | 105,415 |
| 3 | (Carcinoma OR Carcinomas OR Adenocarcinoma OR Adenocarcinomas OR Cancer OR Cancers):ti,ab,kw | 237,524 |
| 4 | #2 AND #3 | 13,850 |
| 5 | (Hypernephroma OR Hypernephromas OR “Grawitz Tumor”):ti,ab,kw | 7 |
| 6 | #1 OR #4 OR #5 | 13,853 |
| 7 | (Pembrolizumab OR Lambrolizumab):ti,ab,kw | 3,804 |
| 8 | (SCH-900475 OR SCH900475 OR “SCH 900475”):ti,ab,kw | 12 |
| 9 | (MK-3475 OR MK3475 OR “MK 3475”):ti,ab,kw | 421 |
| 10 | (Keytruda):ti,ab,kw | 252 |
| 11 | #7 OR #8 OR #9 OR #10 | 3,830 |
| 12 | MeSH descriptor: [Cost-Benefit Analysis] explode all trees | 11,755 |
| 13 | (Cost-Effectiveness OR “Cost Effectiveness”):ti,ab,kw | 28,030 |
| 14 | (Cost-Utility OR “Cost Utility”):ti,ab,kw | 2,866 |
| 15 | (“Economic Evaluation”):ti,ab,kw | 5,453 |
| 16 | #12 OR #13 OR #14 OR #15 | 33,696 |
| 17 | #6 AND #11 AND #16 | 15 |

Supplementary Table 4. Technology appraisals included the study

| **Reference** | **Stage** | **Indication** | **Intervention** | **Comparator (for base case)** | **Recommendation date** |
| --- | --- | --- | --- | --- | --- |
| **NICE** | | | | | |
| **Melanoma** |  |  |  |  |  |
| TA766 | Early | Adjuvant treatment of completely resected stage 3 melanoma | Pembrolizumab | Routine surveillance | 2 February 2022 |
| TA837 | Early | Adjuvant treatment of resected stage 2B or 2C melanoma | Pembrolizumab | Routine surveillance | 26 October 2022 |
| TA357 | Advanced | Advanced melanoma after disease progression with Ipilimumab | Pembrolizumab | Best supportive care | 7 October 2015 |
| TA366 | Advanced | Advanced melanoma not previously treated with Ipilimumab | Pembrolizumab | - BRAF wild type: Ipilimumab - BRAF positive: Ipilimumab, Vemurafenib, Dabrafenib | 25 November 2015 |
| **TNBC** |  |  |  |  |  |
| TA851 | Early | Neoadjuvant and adjuvant treatment of triple-negative early or locally advanced breast cancer | Pembrolizumab+chemotherapy^a^ | Chemotherapy^b^ | 14 December 2022 |
| TA801 | Advanced | Untreated, triple-negative, locally recurrent unresectable or metastatic breast cancer | Pembrolizumab+chemotherapy | Taxane(Paclitaxel, Docetaxel) | 29 June 2022 |
| **RCC** |  |  |  |  |  |
| TA830 | Early | Adjuvant treatment of renal cell carcinoma | Pembrolizumab | Established clinical management | 19 October 2022 |
| TA650 | Advanced | Untreated advanced renal cell carcinoma | Pembrolizumab+Axitinib | Tivozanib, Pazopanib, Cabozantinib | Not Recommended |
| TA858 | Advanced | Untreated advanced renal cell carcinoma | Pembrolizumab+Lenvatinib | Tivozanib, Pazopanib, Sunitinib | 11 January 2023 |
| **CDA-AMC** | | | | | |
| **Melanoma** |  |  |  |  |  |
| PC0168 | Early | Adjuvant treatment of stage 3 melanoma following resection | Pembrolizumab | Observation | 1 August 2019 |
| PC0286 | Early | Adjuvant treatment of adult and pediatric (12 years and older) patients with stage 2B or 2C melanoma following complete resection | Pembrolizumab | Observation | 4 November 2022 |
| PC0058 | Advanced | Unresectable or metastatic melanoma | Pembrolizumab | - Ipilimumab-naive: Ipilimumab - Ipilimumab-refractory: Best supportive care | 16 November 2015 |
| **TNBC** |  |  |  |  |  |
| PC0279 | Early | Treatment of adult patients with high-risk early stage triple-negative breast cancer in combination with chemotherapy as neoadjuvant treatment, and then continued as monotherapy as adjuvant treatment after surgery | Pembrolizumab+chemotherapy^a^ | Chemotherapy^b^ | 31 August 2022 |
| PC0295 | Advanced | Treatment of adult patients with locally recurrent unresectable or metastatic triple-negative breast cancer who have not received prior chemotherapy for metastatic disease and whose tumours express PD-L1 (CPS≥10) as determined by a validated test | Pembrolizumab+chemotherapy | Chemotherapy | 6 January 2023 |
| **RCC** |  |  |  |  |  |
| PC0273 | Early | Adjuvant treatment of adult patients with RCC at intermediate-high or high risk of recurrence following nephrectomy, or following nephrectomy and resection of metastatic lesions | Pembrolizumab | Routine surveillance | 29 September 2022 |
| PC0185 | Advanced | Advanced renal cell carcinoma | Pembrolizumab+Axitinib | Sunitinib, Pazopanib, Nivolumab+ Ipilimumab | 2 April 2020 |
| PC0268 | Advanced | Advanced or metastatic renal cell carcinoma with no prior systemic therapy for metastatic renal cell carcinoma | Pembrolizumab+Lenvatinib | Pembrolizumab+Axitinib, Sunitinib, Pazopanib, Nivolumab+Ipilimumab | 23 June 2022 |
| **PBAC^c^** | | | | | |
| **Melanoma** |  |  |  |  |  |
| November 2018 & July 2019 | Early | Adjuvant treatment for completely resected stage 3 melanoma | Pembrolizumab | Observation | March 2020 |
| March 2015 & March 2016 & November 2016 | Advanced | Unresectable stage 3 or stage 4 metastatic melanoma | Pembrolizumab | Ipilimumab | March 2015 |
| **TNBC** |  |  |  |  |  |
| March 2023 & July 2023 | Early | High risk early stage triple negative breast cancer (TNBC) patients who have not had prior systemic therapy administered for newly diagnosed, locally advanced, centrally confirmed TNBC | Pembrolizumab+chemotherapy^a^ | Chemotherapy^b^ | July 2023 |
| March 2023 | Advanced | Patients with Locally recurrent unresectable or metastatic triple negative breast cancer whose tumours express PD-L1 (CPS≥10) as determined by validated CPS testing and who have not received prior chemotherapy for metastatic disease | Pembrolizumab+chemotherapy | Chemotherapy | March 2023 |
| CPS: combined positive score, PD-L1: programmed cell death-ligand 1  ^a^ Pembrolizumab was used in combination with chemotherapy when administered as neoadjuvant treatment and was given as monotherapy when used as adjuvant therapy following surgery.  ^b^ The comparator was neoadjuvant chemotherapy, and no active treatment as adjuvant setting.  ^c^ PBAC appraisals are referenced by the month and year of the evaluation meeting. | | | | | |

Supplementary Table 5. Published articles included the study

| **Study** | **Country** | **Intervention** | **Comparator** | **Model type** | **Health states** | **Time horizon** | **Perspective** | **Cost-effectiveness** | **Funding source** |
| --- | --- | --- | --- | --- | --- | --- | --- | --- | --- |
| **Early** | | | | | | | | | |
| **Melanoma** |  |  |  |  |  |  |  |  |  |
| Bensimon 2019 | US | Pembrolizumab | Observation | Markov model | RF, LR, DM, Death | 46yrs | Health system | Cost-effective | Industry |
| Bensimon 2020 | US | Pembrolizumab | Observation, Ipilimumab, Dabrafenib+Trametinib | Markov model | RF, LR, DM, Death | Lifetime  (w/o period) | Health system | Cost-effective | Industry |
| Favre-Bulle 2023 | Switzerland | Pembrolizumab | Observation | Markov model | RF, LR, DM, Death | 40.7yrs | Payer | Cost-effective | Industry |
| Lopez-Vinueza 2023 | Colombia | Pembrolizumab | Watchful waiting | Markov model | RF, LR, DM, Death | 46yrs | Payer | Cost-effective | Industry |
| Mojtahed 2021 | US | Pembrolizumab | No treatment | Microsimulation model | RF, LR, DM, Death | 19.4yrs | Medicare | Cost-effective | University |
| Mulder 2021 | Netherland | Pembrolizumab | Routine surveillance | Markov model | NED, RPD, Death | Lifetime  (w/o period) | Societal | Cost-effective | NA |
| Standage 2021 | Portland | Pembrolizumab | Observation | Markov model | RF, LR, DM, Death | 5yrs | Societal | Not cost-effective | NA |
| Wurcel 2021 | Argentina | Pembrolizumab | Watchful waiting | Markov model | RF, LR, DM, Death | 46yrs | Healthcare system | Cost-effective | Industry |
| Zhang 2023 | US | Pembrolizumab | Observation | Markov model | RF, LR, DM, Death | Lifetime  (w/o period) | Health sector | Cost-effective | Industry |
| **TNBC** |  |  |  |  |  |  |  |  |  |
| Favre-Bulle 2024 | Switzerland | Pembrolizumab+chemotherapy | Chemotherapy | Markov model | EF, LR, DM, Death | 51yrs | Payer | Cost-effective | Industry |
| Huang 2023 | US | Pembrolizumab+chemotherapy | Chemotherapy | Markov model | EF, LR, DM, Death | 51yrs | Payer | Cost-effective | Industry |
| Kwong 2024 | Hong Kong | Pembrolizumab+chemotherapy | Chemotherapy | Markov model | EF, LR, DM, Death | 32yrs | Payer | Cost-effective | Industry |
| Pollinger 2025 | Egypt | Pembrolizumab+chemotherapy | Chemotherapy | Markov model | EF, LR, DM, Death | 51yrs | Societal | Cost-effective | Industry |
| **RCC** |  |  |  |  |  |  |  |  |  |
| Lai 2023 | US | Pembrolizumab | Routine surveillance, Sunitinib | Markov model | DF, LR, DM, Death | Lifetime  (w/o period) | Health sector | Cost-effective | Industry |
| Schur 2024 | Switzerland | Pembrolizumab | Routine observation | Markov model | DF, LR, DM, Death | Lifetime  (w/o period) | Payer | Cost-effective | Industry |
| Sharma 2023 | US | Pembrolizumab | Observation | Markov model | No progression, Minor toxicity, Major toxicity, Pembrolizumab discontinuation, Cancer progression, Death | 5yrs | Payer | Not cost-effective | Not funded |
| **Advanced** | | | | | | | | | |
| **Melanoma** |  |  |  |  |  |  |  |  |  |
| Ball 2023 | Canada | Pembrolizumab | Ipilimumab | PSM | PF, PD, Death | 20yrs | Payer | NA^c^ | Not funded |
| Bashari 2024 | Iran | Pembrolizumab | Other immunotherapy^a^ | PSM | PF, PD, Death | 30yrs | Payer | Not cost-effective | University |
| Heine 2024 | Netherlands | Pembrolizumab | Ipilimumab | PSM | PF, PD, Death | 30yrs | Healthcare | Not cost-effective | Not funded |
| Loong 2020 | Hong Kong | Pembrolizumab | Dacarbazine | PSM | PF, PD, Death | 30yrs | Payer | Cost-effective | Industry |
| Miguel 2017 | Portugal | Pembrolizumab | Ipilimumab | PSM | PF, PD, Death | 40yrs | Health Service | Cost-effective | Industry |
| Tang 2022 | China | Pembrolizumab | Paclitaxel+Carboplatin | PSM | PF, PD, Death | 20yrs | Healthcare system | Cost-effective | Industry |
| Wang 2017 | US | Pembrolizumab | Ipilimumab | PSM | PF, PD, Death | 20yrs | Health system | Cost-effective | Industry |
| Wu 2020 | US | Pembrolizumab | Ipilimumab, Nivolumab, Nivolumab+Ipilimumab,  4 BRAF guided strategy | PSM | PF, PD, Death | Lifetime  (w/o period) | Payer | Cost-effective | NA |
| **TNBC** |  |  |  |  |  |  |  |  |  |
| Huang 2022 | US | Pembrolizumab+chemotherapy | Chemotherapy, Atezolizumab+chemotherapy | PSM | PF, PD, Death | 20yrs | Payer | Cost-effective | Industry |
| Zhu 2023 | China | Pembrolizumab+chemotherapy | Chemotherapy | Markov model | PF, PD, Death | 10yrs | Healthcare system | Not cost-effective | NA |
| **RCC** |  |  |  |  |  |  |  |  |  |
| Bensimon 2020 | US | Pembrolizumab+Axitinib | - Overall population: Sunitinib, Pazopanib, Avelumab+Axitinib - IMDC intermediate/poor risk group: Sunitinib, Cabozantinib, Nivolumab+ Ipilimumab | PSM | PF, PD, Death | 39yrs | Payer | Cost-effective | Industry |
| Chan 2022 | US | Pembrolizumab+Axitinib | Nivolumab+Ipilimumab, Sunitinib | Markov model | PF, PD, Death | 20yrs | Health system | Not cost-effective | NA |
| Chen 2019 | China | Pembrolizumab+Axitinib | Sunitinib | Markov model | PF, PD, Death | Lifetime  (w/o period) | Healthcare | Not cost-effective | Government |
| Ding 2021 | China | Pembrolizumab+Axitinib | Sunitinib | Markov model | PF, PD, Death | Lifetime  (w/o period) | Payer | Cost-effective | Not funded |
| Gupta 2023 | India | Pembrolizumab+Lenvatinib | Nivolumab+Ipilimumab^b^ | Markov model | PF, PD, Death | Lifetime  (w/o period) | Societal | Not cost-effective | Government |
| Heine 2024 | Netherlands | Pembrolizumab+Axitinib | Sunitinib | PSM | PF, PD, Death | 30yrs | Healthcare | Not cost-effective | Not funded |
| Shay 2021 | US | Pembrolizumab+Axitinib | Nivolumab+Ipilimumab, Avelumab+Axitinib | Markov model | PF, PD, Death | 10yrs | Payer | Both^d^ | University |
| Su 2020 | China | Pembrolizumab+Axitinib | Sunitinib | Markov model | PF, PD, Death | 10yrs | Payer | Not cost-effective | Not funded |
| Wang 2022 | China | Pembrolizumab+Lenvatinib | Sunitinib | PSM | PF, PD, Death | 5yrs | Health system | Not cost-effective | Not funded |
| Watson 2020 | US | Pembrolizumab+Axitinib | Nivolumab+Ipilimumab | Microsimulation model | PF, PD, Death | NA | Healthcare sector | Not cost-effective | NA |
| Xander 2023 | Netherlands | Pembrolizumab+Axitinib | Sunitinib | PSM | PF, PD, Death | 15yrs | Societal | Not cost-effective | Not funded |
| Yoo 2023 | US | Pembrolizumab+Axitinib,  Pembrolizumab+Lenvatinib | Nivolumab+Ipilimumab | PSM | PF, PD, Death | 10yrs | Payer | Not cost-effective | University |
| Zheng 2025 | China | Pembrolizumab+Lenvatinib | Sunitinib, Everolimus+Lenvatinib | Markov model | PF, PD, Death | 10yrs | Healthcare system | Not cost-effective | University |
| Zhu 2020 | China | Pembrolizumab+Axitinib | Sunitinib | Markov model | PF, PD, Death | 20yrs | Payer | Not cost-effective | Government |
| Zhu 2023 | China | Pembrolizumab+Lenvatinib | Sunitinib | Markov model | PF, PD, Death | 20yrs | Payer | Cost-effective | Not funded |
| DF: disease-free, DM: distant metastasis, EF: event-free, LR: local recurrence, PD: progressed disease, PF: progression-free, PSM: partitioned survival model  ^a^ Included temozolomide, nivolumab, nivolumab+ipilimumab.  ^b^ Gupta 2023 was a comparative study of four treatments: sunitinib, pazopanib, pembrolizumab+lenvatinib, and nivolumab+ipilimumab. The results of pembrolizumab+lenvatinib were only presented in comparison with nivolumab+ipilimumab.  ^c^ The conclusion on cost-effectiveness was not provided.  ^d^ It was not cost-effective when compared to nivolumab+ipilimumab, but it was cost-effective(dominant) when compared to avelumab+axitinib | | | | | | | | | |

Supplementary Table 6. Appraisal of the included articles against CHEERS checklist

| **Study** | **CHEERS 2022 item** | | | | | | | | | | | | | | | | | | | | | | | | | | | | |
| --- | --- | --- | --- | --- | --- | --- | --- | --- | --- | --- | --- | --- | --- | --- | --- | --- | --- | --- | --- | --- | --- | --- | --- | --- | --- | --- | --- | --- | --- |
|  | **1** | **2** | **3** | **4** | **5** | **6** | **7** | **8** | **9** | **10** | **11** | **12** | **13** | **14** | **15** | **16** | **17** | **18** | **19** | **20** | **21** | **22** | **23** | **24** | **25** | **26** | **27** | **28** |  |
| **Early Melanoma** |  |  |  |  |  |  |  |  |  |  |  |  |  |  |  |  |  |  |  |  |  |  |  |  |  |  |  |  |  |
| Bensimon 2019 | R | R | R | NR | R | R | R^a^ | R | R | R | R | R | R | R | R | R^a^ | R | NR | NA | R | NA | R | R | R | NA | R | R | R |  |
| Bensimon 2020 | R | R | R | NR | R | R | R | R | R^a^ | R | R | R | R | R | R | R | R | NR | NA | R | NA | R | R | R | NA | R | R | R |  |
| Favre-Bulle 2023 | R | R | R | NR | R | R | R | R | R | R | R | R | R | R | R | R^a^ | R | NR | NA | R | NA | R | R | R | NA | R | R | R |  |
| Lopez-Vinueza 2023 | R | R | R | NR | R | R | R | R | R | R | R | R | R | R | R | R^a^ | R | NR | NA | R | NA | R | R | R | NA | R | R | R |  |
| Mojtahed 2021 | R | R | R | NR | R | R | R^a^ | R^a^ | R | R^a^ | R | R | R | R | R | R^a^ | R | NR | NA | R | NA | R | R | R | NA | R | R | R |  |
| Mulder 2021 | R | R | R | NR | R | R | R | R^a^ | R^a^ | R | R | R | R | R | R | R^a^ | R | NR | NA | R | NA | R | R | R | NA | R | R | R |  |
| Standage 2021 | NR | R | R | NR | R | R | R^a^ | R | R^a^ | R | R | R | R | R | R | R^a^ | R | NR | NA | R | NA | R | R | R | NA | R | NR | R |  |
| Wurcel 2021 | R | R | R | NR | R | R | R^a^ | R^a^ | R | R | R | R | R | R | R | R^a^ | R | R | NA | R | NA | R | R | R | NA | R | R | R |  |
| Zhang 2023 | R | R | R | NR | R | R | R^a^ | R^a^ | R^a^ | R | R | R | R | R | R | R^a^ | R | NR | NA | R | NA | R | R | R | NA | R | R | R |  |
| **TNBC** |  |  |  |  |  |  |  |  |  |  |  |  |  |  |  |  |  |  |  |  |  |  |  |  |  |  |  |  |  |
| Favre-Bulle 2024 | R | R | R | NR | R | R | R | R | R | R | R | R | R | R | R | R^a^ | R | NR | NA | R | NA | R | R | R | NA | R | R | R |  |
| Huang 2023 | R | R | R | NR | R | R | R | R^a^ | R^a^ | R | R | R | R | R | R | R^a^ | R | NR | NA | R | NA | R | R | R | NA | R | R | R |  |
| Kwong 2024 | R | R | R | NR | R | R | R | R^a^ | R | R | R | R | R | R | R | R^a^ | R | NR | NA | R | NA | R | R | R | NA | R | R | R |  |
| Pollinger 2025 | R | R | R | NR | R | R | R^a^ | R^a^ | R^a^ | R | R | R | R | R | R | R | R | NR | NA | R | NA | R | R | R | NA | R | R | R |  |
| **RCC** |  |  |  |  |  |  |  |  |  |  |  |  |  |  |  |  |  |  |  |  |  |  |  |  |  |  |  |  |  |
| Lai 2023 | R | R | R | NR | R | R | R^a^ | R^a^ | R^a^ | R | R | R | R | R | R | R^a^ | R | NR | NA | R | NA | R | R | R | NA | R | R | R |  |
| Schur 2024 | R | R | R | NR | R | R | R^a^ | R^a^ | R | R^a^ | R | R | R | R | R | R^a^ | R | NR | NA | R | NA | R | R | R | NA | R | R | R |  |
| Sharma 2023 | R | R | R | NR | R | R | R^a^ | R^a^ | R^a^ | R^a^ | R | R | R | R | R | R^a^ | R | NR | NA | R | NA | R | R | R | NA | R | R | R |  |
| **Advanced Melanoma** |  |  |  |  |  |  |  |  |  |  |  |  |  |  |  |  |  |  |  |  |  |  |  |  |  |  |  |  |  |
| Ball 2023 | NR | R | R | NR | R | R | R^a^ | R | R^a^ | R | R | R | R | R | R | R^a^ | R | NR | NA | R | NA | R | R | R | NA | R | R | R |  |
| Bashari 2024 | R | R | R | NR | R | R | R | R^a^ | R | R^a^ | R | R | R | R | R | R | R | NR | NA | R | NA | R | R | R | NA | R | R | R |  |
| Heine 2024 | NR | R | R | NR | R | R | R | R^a^ | R^a^ | R | R | R | R | R | R | R^a^ | R | NR | NA | R | NA | R | R | R | NA | R | R | R |  |
| Loong 2020 | R | R | R | NR | R | R | R^a^ | R^a^ | R | R^a^ | R | R | R | R | R | R^a^ | R | NR | NA | R | NA | R | R | R | NA | R | R | R |  |
| Miguel 2017 | R | R | R | NR | R | R | R^a^ | R^a^ | R | R | R | R | R | R | R | R^a^ | R | NR | NA | R | NA | R | R | R | NA | R | R | R |  |
| Tang 2022 | R | R | R | NR | R | R | R^a^ | R^a^ | R | R | R | R | R | R | R | R^a^ | R | NR | NA | R | NA | R | R | R | NA | R | R | R |  |
| Wang 2017 | R | R | R | NR | R | R | R^a^ | R^a^ | R^a^ | R^a^ | R | R | R | R | R | R^a^ | R | NR | NA | R | NA | R | R | R | NA | R | R | R |  |
| Wu 2020 | R | R | R | NR | R | R | R^a^ | R^a^ | R^a^ | R | R | R | R | R | R | R^a^ | R | R | NA | R | NA | R | R | R | NA | R | R | R |  |
| **TNBC** |  |  |  |  |  |  |  |  |  |  |  |  |  |  |  |  |  |  |  |  |  |  |  |  |  |  |  |  |  |
| Huang 2022 | R | R | R | NR | R | R | R^a^ | R^a^ | R | R | R | R | R | R | R | R | R | R | NA | R | NA | R | R | R | NA | R | R | R |  |
| Zhu 2023 | R | R | R | NR | R | R | R^a^ | R^a^ | R^a^ | R | R | R | R | R | R | R^a^ | R | NR | NA | R | NA | R | R | R | NA | R | NR | NR |  |
| **RCC** |  |  |  |  |  |  |  |  |  |  |  |  |  |  |  |  |  |  |  |  |  |  |  |  |  |  |  |  |  |
| Bensimon 2020 | R | R | R | NR | R | R | R | R^a^ | R | R | R | R | R | R | R | R | R | NR | NA | R | NA | R | R | R | NA | R | R | R |  |
| Chan 2022 | R | R | R | NR | R | R | R^a^ | R^a^ | R^a^ | R^a^ | R | R | R | R | R | R^a^ | R | NR | NA | R | NA | R | R | R | NA | R | NR | R |  |
| Chen 2019 | R | R | R | NR | R | R | R^a^ | R^a^ | R^a^ | R^a^ | R | R | R | R | R | R^a^ | R | R | NA | R | NA | R | R | R | NA | R | R | R |  |
| Ding 2021 | R | R | R | NR | R | R | R^a^ | R^a^ | R^a^ | R^a^ | R | R | R | R | R | R^a^ | R | R | NA | R | NA | R | R | R | NA | R | NR | R |  |
| Gupta 2023 | R | R | R | NR | R | R | R | R | R | R | R | R | R | R | R | R^a^ | R | NR | NA | R | NA | R | R | R | NA | R | R | R |  |
| Heine 2024 | NR | R | R | NR | R | R | R | R^a^ | R^a^ | R | R | R | R | R | R | R^a^ | R | NR | NA | R | NA | R | R | R | NA | R | R | R |  |
| Shay 2021 | R | R | R | NR | R | R | R^a^ | R^a^ | R^a^ | R^a^ | R | R | R | R | R | R^a^ | R | NR | NA | R | NA | R | R | R | NA | R | R | R |  |
| Su 2020 | R | R | R | NR | R | R | R | R^a^ | R | R^a^ | R | R | R | R | R | R^a^ | R | R | NA | R | NA | R | R | R | NA | R | R | R |  |
| Wang 2022 | R | R | R | NR | R | R | R^a^ | R^a^ | R^a^ | R^a^ | R | R | R | R | R | R | R | R | NA | R | NA | R | R | R | NA | R | R | R |  |
| Watson 2020 | R | R | R | NR | R | R | R^a^ | R^a^ | NR | R^a^ | R | R | R | R | R | R^a^ | R | NR | NA | R | NA | R | R | R | NA | R | NR | R |  |
| Xander 2023 | R | R | R | NR | R | R | R^a^ | R^a^ | R^a^ | R | R | R | R | R | R | R | R | R | NA | R | NA | R | R | R | NA | R | NR | R |  |
| Yoo 2023 | R | R | R | NR | R | R | R | R^a^ | R | R^a^ | R | R | R | R | R | R | R | NR | NA | R | NA | R | R | R | NA | R | R | R |  |
| Zheng 2025 | R | R | R | NR | R | R | R^a^ | R^a^ | R^a^ | R^a^ | R | R | R | R | R | R^a^ | R | NR | NA | R | NA | R | R | R | NA | R | R | R |  |
| Zhu 2020 | R | R | R | NR | R | R | R^a^ | R^a^ | R^a^ | R^a^ | R | R | R | R | R | R^a^ | R | R | NA | R | NA | R | R | R | NA | R | R | R |  |
| Zhu 2023 | R | R | R | NR | R | R | R^a^ | R^a^ | R | R^a^ | R | R | R | R | R | R^a^ | R | R | NA | R | NA | R | R | R | NA | R | R | R |  |
| CHEERS item no. 1: Title, 2: Abstract, 3: Background and objectives, 4: Health economic analysis plan, 5: Study population, 6: Setting and location, 7: Comparators, 8: Perspective, 9: Time horizon, 10: Discount rate, 11: Selection of outcomes, 12: Measurement of outcomes, 13: Valuation of outcomes, 14: Measurement and valuation of resources and costs, 15: Currency, price date, and conversion, 16: Rationale and description of model, 17: Analytics and assumptions, 18: Characterising heterogeneity, 19: Characterising distributional effects, 20: Characterising uncertainty, 21: Approach to engagement with patients and others affected by the study, 22: Study parameters, 23: Summary of main results, 24: Effect of uncertainty, 25: Effect of engagement with patients and others affected by the study, 26: Study findings, limitations, generalisability, and current knowledge, 27: Source of funding, 28: Conflicts of interest  R: Reported, NR: Not reported, NA: Not applicable  ^a^ Reported but the reason was not reported | | | | | | | | | | | | | | | | | | | | | | | | | | | | |  |
